# Supplementary material for: Heavy metal contamination and ecological risk assessment of the agricultural soil in Shanxi Province, China
Source: R Soc Open Sci. 2020 Oct 7;7(10):200538. doi: 10.1098/rsos.200538 (PMC7657894; doi:10.1098/rsos.200538)
Supplement: Supporting Information From: Heavy metals contamination and ecological risk assessment of the agricultural soil in Shanxi Province, China [file rsos200538supp1.doc]

**Supporting Information**

# MANUSCRIPT TITLE: Heavy metals contamination and ecological risk assessment of the agricultural soil in Shanxi Province, China

**AUTHORS:** Hongxue Qi, Bingqing Zhao, Lihong Li*, Xiuling Chen, Jing An, Xiuping Liu

**ADDRESS:** College of Chemistry and Chemical Engineering, Jinzhong University, Jinzhong 030619, Shanxi, China

E-MAIL: lily@jzxy.edu.cn

**JOURNAL:** *Royal Society Open Science*

**Figure:** 1

**TABLES:** 5

**NO. OF PAGES:** 9


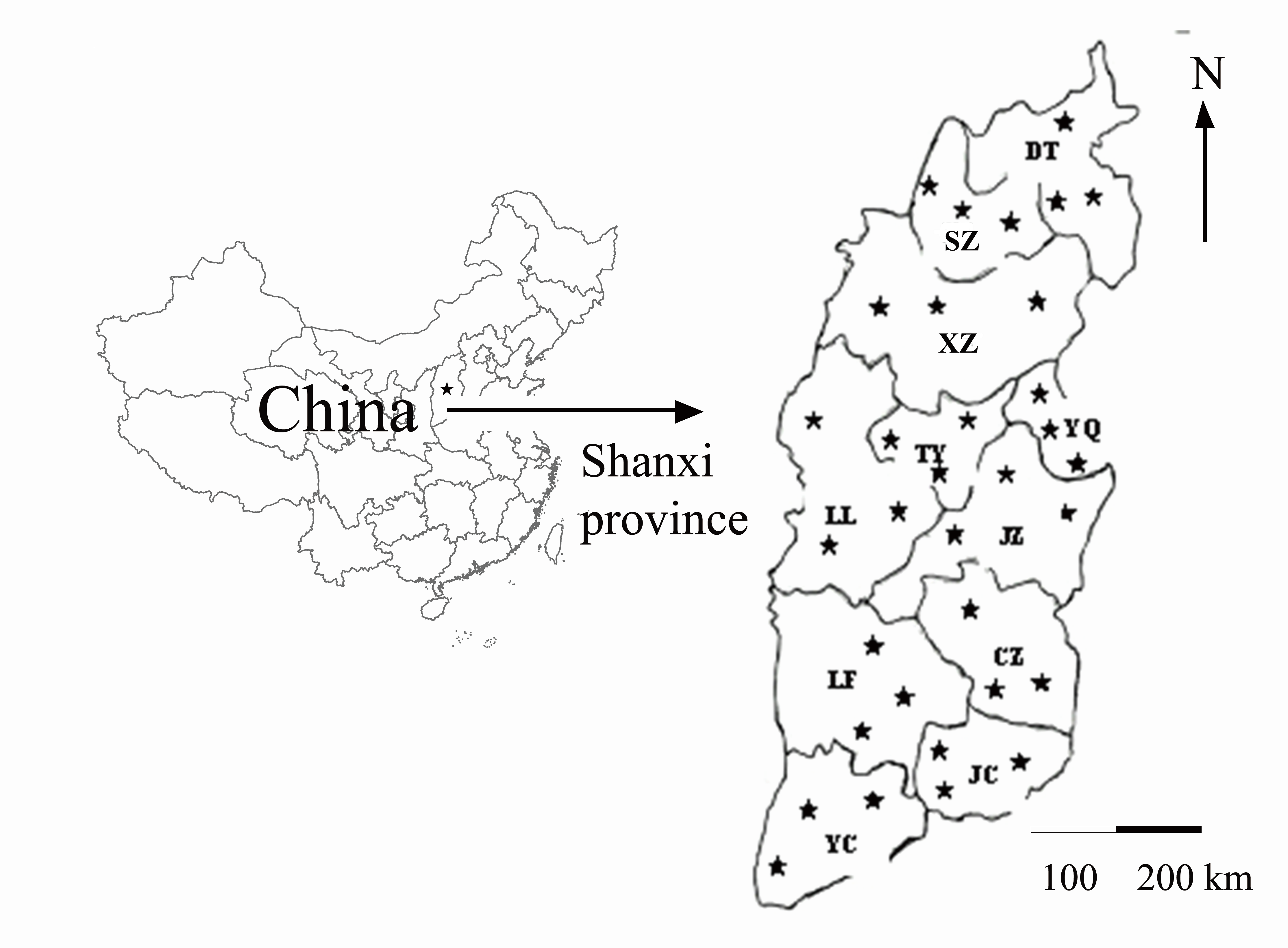


**Figure S1** Locations of the sampling sites in Shanxi province, China (Previously reported in same samples for PAHs analysis) [1] (DT: Datong, SZ: Shuozhou, XZ: Xinzhou, TY: Taiyuan, YQ: Yangquan, LL: Lvliang, JZ: Jinzhong, LF: Linfen, CZ: Changzhi, YC: Yuncheng, JC: Jincheng)

**Table S1 Coal production and consumption (million tons) in Shanxi Province and China, respectively.**

| Year |  | Coal production |  |  |  | Coal consumption |  |
| --- | --- | --- | --- | --- | --- | --- | --- |
|  | Shanxi | China | % |  | Shanxi | China | % |
| 2010 | 629.78 | 2378.39 | 26.5 |  | 281.80 | 2495.68 | 11.3 |
| 2011 | 741.47 | 2646.58 | 28.0 |  | 308.96 | 2717.04 | 11.4 |
| 2012 | 776.36 | 2674.93 | 29.0 |  | 310.85 | 2754.65 | 11.3 |
| 2013 | 682.02 | 2705.23 | 25.2 |  | 330.56 | 2809.99 | 11.8 |
| 2014 | 676.26 | 2663.33 | 25.4 |  | 320.56 | 2793.29 | 11.5 |
| 2015 | 715.90 | 2609.86 | 27.4 |  | 294.28 | 2738.49 | 10.8 |
| 2016 | 619.01 | 2415.34 | 25.6 |  | 300.61 | 2702.08 | 11.1 |
| 2017 | 645.44 | 2495.16 | 25.9 |  | 321.71 | 2709.12 | 11.9 |
| References | [2] | [3] |  |  | [4] | [5] |  |

**Table S2 Results of geoaccumulation index (I_geo_) for heavy metals in the surface soil in Shanxi province in 2018 (*n* = 33)**

|  | Sites | As | Cd | Cr | Cu | Hg | Ni | Pb | Zn |
| --- | --- | --- | --- | --- | --- | --- | --- | --- | --- |
| B_n_ ^a^ |  | 9.1 | 0.102 | 55.3 | 22.9 | 0.023 | 29.9 | 14.7 | 63.5 |
| 1 | DT1 | -0.64 | 1.52 | -1.10 | -1.03 | 1.67 | -1.27 | 0.09 | -0.42 |
| 2 | DT2 | -0.77 | 0.52 | -1.61 | -1.55 | 4.48 | -1.68 | -1.51 | -1.53 |
| 3 | DT3 | -1.50 | 0.59 | -0.93 | -0.06 | 3.17 | -0.88 | -1.34 | -0.77 |
| 4 | SZ1 | -0.31 | 0.23 | -1.04 | -1.20 | 3.54 | -1.33 | -1.18 | -0.80 |
| 5 | SZ2 | -0.49 | 0.76 | -1.27 | -1.08 | 2.86 | -1.27 | -1.19 | -0.79 |
| 6 | SZ3 | -1.03 | 0.59 | -1.07 | -0.69 | 2.38 | -1.21 | -1.29 | -0.78 |
| 7 | XZ1 | -0.86 | 0.46 | -1.25 | -0.20 | 2.91 | -1.25 | -0.16 | -0.65 |
| 8 | XZ2 | -0.49 | -0.13 | -1.50 | -1.04 | 2.67 | -1.54 | -1.14 | -0.66 |
| 9 | XZ3 | 0.56 | 1.49 | -0.34 | 0.19 | 2.74 | -0.48 | -0.28 | 0.37 |
| 10 | TY1 | -0.51 | 1.35 | -1.03 | -0.23 | 3.50 | -1.32 | 0.25 | 0.14 |
| 11 | TY2 | -0.31 | 0.23 | -1.05 | -1.20 | 3.54 | -1.34 | -1.18 | -0.81 |
| 12 | TY3 | -0.27 | -0.77 | -1.53 | -1.59 | 2.38 | -1.62 | -1.55 | -1.31 |
| 13 | YQ1 | -0.65 | 0.31 | -1.37 | -0.96 | 3.61 | -1.50 | -0.91 | -0.39 |
| 14 | YQ2 | -0.30 | 0.52 | -1.17 | -0.75 | 2.02 | -1.19 | -0.81 | -0.61 |
| 15 | YQ3 | 0.05 | 1.02 | -1.03 | -0.62 | 1.67 | -1.12 | -1.07 | 0.37 |
| 16 | JZ1 | -0.53 | 0.52 | -1.05 | -0.93 | 3.07 | -1.20 | -0.84 | -0.58 |
| 17 | JZ2 | -0.46 | 0.71 | -1.03 | -0.98 | 2.91 | -1.12 | -0.72 | -0.51 |
| 18 | JZ3 | -0.80 | 0.65 | -1.33 | -1.04 | 2.67 | -1.54 | -0.65 | -0.21 |
| 19 | LL1 | -0.27 | 0.82 | -1.16 | -0.86 | 2.86 | -1.22 | -1.23 | -0.59 |
| 20 | LL2 | -0.21 | 0.46 | -1.27 | -1.01 | 2.86 | -1.27 | -1.14 | -0.18 |
| 21 | LL3 | -0.23 | 0.87 | -1.16 | -0.87 | 3.26 | -1.25 | -1.14 | -0.58 |
| 22 | CZ1 | 0.38 | 1.59 | -0.64 | -0.18 | 1.54 | -0.82 | -0.15 | 0.71 |
| 23 | CZ2 | -0.06 | 2.39 | -1.09 | -0.68 | 2.97 | -1.09 | 0.29 | -0.18 |
| 24 | CZ3 | 0.25 | 1.15 | -0.78 | -0.22 | 2.02 | -0.68 | -0.25 | -0.04 |
| 25 | LF1 | -0.09 | 1.27 | -1.12 | -0.57 | 3.50 | -1.14 | -0.70 | -0.26 |
| 26 | LF2 | 0.26 | 0.87 | -0.98 | -0.36 | 0.18 | -0.95 | -0.72 | -0.36 |
| 27 | LF3 | 0.40 | 1.31 | -0.83 | -0.03 | 2.74 | -0.88 | -0.18 | 0.11 |
| 28 | JC1 | -0.01 | 1.06 | -1.10 | 0.01 | 1.02 | -1.12 | -0.71 | -0.36 |
| 29 | JC2 | 0.56 | 1.71 | -0.52 | -0.04 | 2.61 | -0.55 | -0.34 | 0.08 |
| 30 | JC3 | 0.73 | 1.39 | -0.47 | 0.19 | 3.17 | -0.41 | -0.13 | 0.33 |
| 31 | YC1 | 0.17 | 0.82 | -0.85 | -0.41 | 3.42 | -0.89 | -0.87 | -0.30 |
| 32 | YC2 | 0.66 | 2.69 | -0.17 | 1.10 | 2.46 | -0.54 | 1.62 | 1.28 |
| 33 | YC3 | 0.77 | 2.95 | 0.17 | 1.64 | 0.75 | -0.30 | 1.31 | 1.66 |

^b^ Background: Soil elements background values in Shanxi, China [6].

**Table S3 Concentrations of heavy metals in soil samples (*C*_s_, mg/kg) were collected from the surface soil in Shanxi, China.**

| No. | Sites | pH | As | Cd | Cr | Cu | Hg | Ni | Pb | Zn |
| --- | --- | --- | --- | --- | --- | --- | --- | --- | --- | --- |
| 1 | DT1 | 8.29 | 8.75 | 0.44 | 38.8 | 16.8 | 0.11 | 18.6 | 23.5 | 71.1 |
| 2 | DT2 | 8.58 | 8.00 | 0.22 | 27.2 | 11.7 | 0.77 | 14.0 | 7.73 | 32.9 |
| 3 | DT3 | 8.86 | 4.83 | 0.23 | 43.6 | 33.0 | 0.31 | 24.3 | 8.72 | 55.7 |
| 4 | SZ1 | 8.93 | 11.0 | 0.18 | 40.3 | 15.0 | 0.40 | 17.8 | 9.75 | 54.7 |
| 5 | SZ2 | 7.68 | 9.69 | 0.26 | 34.3 | 16.3 | 0.25 | 18.6 | 9.65 | 55.2 |
| 6 | SZ3 | 8.76 | 6.69 | 0.23 | 39.6 | 21.3 | 0.18 | 19.4 | 9.01 | 55.6 |
| 7 | XZ1 | 8.09 | 7.53 | 0.21 | 34.8 | 30.0 | 0.26 | 18.9 | 19.7 | 60.5 |
| 8 | XZ2 | 7.76 | 9.75 | 0.14 | 29.4 | 16.7 | 0.22 | 15.4 | 10.0 | 60.3 |
| 9 | XZ3 | 8.46 | 20.1 | 0.43 | 65.4 | 39.1 | 0.23 | 32.1 | 18.1 | 123 |
| 10 | TY1 | 7.96 | 9.60 | 0.39 | 40.7 | 29.3 | 0.39 | 18.0 | 26.2 | 105 |
| 11 | TY2 | 8.13 | 11.0 | 0.18 | 40.1 | 14.9 | 0.40 | 17.7 | 9.70 | 54.4 |
| 12 | TY3 | 8.29 | 11.3 | 0.09 | 28.7 | 11.4 | 0.18 | 14.6 | 7.52 | 38.5 |
| 13 | YQ1 | 8.32 | 8.68 | 0.19 | 32.1 | 17.6 | 0.42 | 15.9 | 11.7 | 72.8 |
| 14 | YQ2 | 8.07 | 11.1 | 0.22 | 36.9 | 20.4 | 0.14 | 19.6 | 12.6 | 62.2 |
| 15 | YQ3 | 8.69 | 14.1 | 0.31 | 40.5 | 22.3 | 0.11 | 20.7 | 10.5 | 123 |
| 16 | JZ1 | 8.64 | 9.48 | 0.22 | 40.1 | 18.0 | 0.29 | 19.5 | 12.3 | 63.9 |
| 17 | JZ2 | 7.99 | 9.95 | 0.25 | 40.5 | 17.4 | 0.26 | 20.7 | 13.4 | 67.0 |
| 18 | JZ3 | 7.81 | 7.85 | 0.24 | 33.0 | 16.7 | 0.22 | 15.4 | 14.1 | 82.1 |
| 19 | LL1 | 8.14 | 11.3 | 0.27 | 37.0 | 18.9 | 0.25 | 19.2 | 9.41 | 63.3 |
| 20 | LL2 | 8.37 | 11.8 | 0.21 | 34.4 | 17.1 | 0.25 | 18.6 | 9.99 | 83.8 |
| 21 | LL3 | 8.54 | 11.6 | 0.28 | 37.1 | 18.8 | 0.33 | 18.8 | 10.0 | 63.8 |
| 22 | CZ1 | 8.29 | 17.8 | 0.46 | 53.3 | 30.3 | 0.10 | 25.4 | 19.9 | 156 |
| 23 | CZ2 | 7.53 | 13.1 | 0.80 | 39.1 | 21.5 | 0.27 | 21.1 | 26.9 | 83.9 |
| 24 | CZ3 | 7.83 | 16.2 | 0.34 | 48.4 | 29.5 | 0.14 | 27.9 | 18.5 | 92.9 |
| 25 | LF1 | 8.14 | 12.8 | 0.37 | 38.2 | 23.1 | 0.39 | 20.4 | 13.6 | 79.5 |
| 26 | LF2 | 6.94 | 16.3 | 0.28 | 42.0 | 26.7 | 0.039 | 23.2 | 13.4 | 74.0 |
| 27 | LF3 | 8.51 | 18.0 | 0.38 | 46.7 | 33.7 | 0.23 | 24.4 | 19.5 | 103 |
| 28 | JC1 | 8.05 | 13.6 | 0.32 | 38.8 | 34.5 | 0.07 | 20.6 | 13.5 | 74.1 |
| 29 | JC2 | 8.23 | 20.1 | 0.50 | 57.8 | 33.5 | 0.21 | 30.6 | 17.4 | 101 |
| 30 | JC3 | 8.14 | 22.6 | 0.40 | 60.0 | 39.2 | 0.31 | 33.8 | 20.1 | 120 |
| 31 | YC1 | 8.37 | 15.4 | 0.27 | 45.9 | 25.9 | 0.37 | 24.2 | 12.1 | 77.1 |
| 32 | YC2 | 7.56 | 21.5 | 0.99 | 73.8 | 73.8 | 0.19 | 30.8 | 67.6 | 231 |
| 33 | YC3 | 8.61 | 23.2 | 1.18 | 93.1 | 107 | 0.058 | 36.4 | 54.7 | 300 |

**Table S4 Percentage of potential ecological risk for heavy metals in the surface soil in Shanxi (*n* = 33)**

|  | Sites | As | Cd | Cr | Cu | Hg | Ni | Pb | Zn | RI (Risk indices) |
| --- | --- | --- | --- | --- | --- | --- | --- | --- | --- | --- |
| *T_i_*^a^ |  | 10 | 30 | 2 | 5 | 40 | 5 | 5 | 1 |  |
| 1 | DT1 | 9.62 | 129 | 1.40 | 3.67 | 191 | 3.11 | 7.99 | 1.12 | 348 |
| 2 | DT2 | 8.79 | 64.7 | 0.98 | 2.55 | 1339 | 2.34 | 2.63 | 0.52 | 1422 |
| 3 | DT3 | 5.31 | 67.6 | 1.58 | 7.21 | 539 | 4.06 | 2.97 | 0.88 | 629 |
| 4 | SZ1 | 12.1 | 52.9 | 1.46 | 3.28 | 696 | 2.98 | 3.32 | 0.86 | 773 |
| 5 | SZ2 | 10.65 | 76.5 | 1.24 | 3.56 | 435 | 3.11 | 3.28 | 0.87 | 534 |
| 6 | SZ3 | 7.35 | 67.6 | 1.43 | 4.65 | 313 | 3.24 | 3.06 | 0.88 | 401 |
| 7 | XZ1 | 8.27 | 61.8 | 1.26 | 6.55 | 452 | 3.16 | 6.70 | 0.95 | 541 |
| 8 | XZ2 | 10.71 | 41.2 | 1.06 | 3.65 | 383 | 2.58 | 3.40 | 0.95 | 446 |
| 9 | XZ3 | 22.1 | 126 | 2.37 | 8.54 | 400 | 5.37 | 6.16 | 1.94 | 573 |
| 10 | TY1 | 10.55 | 114.7 | 1.47 | 6.40 | 678 | 3.01 | 8.91 | 1.65 | 825 |
| 11 | TY2 | 12.1 | 52.9 | 1.45 | 3.25 | 696 | 2.96 | 3.30 | 0.86 | 773 |
| 12 | TY3 | 12.4 | 26.5 | 1.04 | 2.49 | 313 | 2.44 | 2.56 | 0.61 | 361 |
| 13 | YQ1 | 9.54 | 55.9 | 1.16 | 3.84 | 730 | 2.66 | 3.98 | 1.15 | 809 |
| 14 | YQ2 | 12.2 | 64.7 | 1.33 | 4.45 | 243 | 3.28 | 4.29 | 0.98 | 335 |
| 15 | YQ3 | 15.5 | 91.2 | 1.46 | 4.87 | 191 | 3.46 | 3.57 | 1.94 | 313 |
| 16 | JZ1 | 10.42 | 64.7 | 1.45 | 3.93 | 504 | 3.26 | 4.18 | 1.01 | 593 |
| 17 | JZ2 | 10.9 | 73.5 | 1.46 | 3.80 | 452 | 3.46 | 4.56 | 1.06 | 551 |
| 18 | JZ3 | 8.63 | 70.6 | 1.19 | 3.65 | 383 | 2.58 | 4.80 | 1.29 | 475 |
| 19 | LL1 | 12.4 | 79.4 | 1.34 | 4.13 | 435 | 3.21 | 3.20 | 1.00 | 539 |
| 20 | LL2 | 13.0 | 61.8 | 1.24 | 3.73 | 435 | 3.11 | 3.40 | 1.32 | 522 |
| 21 | LL3 | 12.7 | 82.4 | 1.34 | 4.10 | 574 | 3.14 | 3.40 | 1.00 | 682 |
| 22 | CZ1 | 19.6 | 135 | 1.93 | 6.62 | 174 | 4.25 | 6.77 | 2.46 | 351 |
| 23 | CZ2 | 14.4 | 235 | 1.41 | 4.69 | 470 | 3.53 | 9.15 | 1.32 | 739 |
| 24 | CZ3 | 17.8 | 100.0 | 1.75 | 6.44 | 243 | 4.67 | 6.29 | 1.46 | 382 |
| 25 | LF1 | 14.1 | 108.8 | 1.38 | 5.04 | 678 | 3.41 | 4.63 | 1.25 | 817 |
| 26 | LF2 | 17.9 | 82.4 | 1.52 | 5.83 | 67.8 | 3.88 | 4.56 | 1.17 | 185 |
| 27 | LF3 | 19.8 | 111.8 | 1.69 | 7.36 | 400 | 4.08 | 6.63 | 1.62 | 553 |
| 28 | JC1 | 14.9 | 94.1 | 1.40 | 7.53 | 122 | 3.44 | 4.59 | 1.17 | 249 |
| 29 | JC2 | 22.1 | 147 | 2.09 | 7.31 | 365 | 5.12 | 5.92 | 1.59 | 556 |
| 30 | JC3 | 24.8 | 117.6 | 2.17 | 8.56 | 539 | 5.65 | 6.84 | 1.89 | 707 |
| 31 | YC1 | 16.9 | 79.4 | 1.66 | 5.66 | 643 | 4.05 | 4.12 | 1.21 | 757 |
| 32 | YC2 | 23.6 | 291 | 2.67 | 16.1 | 330 | 5.15 | 23.0 | 3.64 | 696 |
| 33 | YC3 | 25.5 | 347 | 3.37 | 23.4 | 100.9 | 6.09 | 18.6 | 4.72 | 530 |
| Minimum |  | 5.31 | 26.5 | 0.98 | 2.49 | 67.8 | 2.34 | 2.56 | 0.52 | 185 |
| Median |  | 12.4 | 79.4 | 1.45 | 4.69 | 435 | 3.28 | 4.56 | 1.17 | 551 |
| Maximum |  | 25.5 | 347 | 3.37 | 23.4 | 1339 | 6.09 | 23.0 | 4.72 | 1422 |
| Mean |  | 13.8 | 99.5 | 1.55 | 5.83 | 429 | 3.58 | 5.65 | 1.37 | 563 |

^a^ *T_i_*: toxicity response coefficient for the metal *i*.

**Detailed description of metals analyses**

Soil samples were analyzed for eight metals, including As, Cd, Cr, Cu, Hg, Ni, Pb, and Zn. Multi-element standards were obtained from Agilent, soil standard reference material (GBW07408, GSS-8) was purchased from the Center of National Standard Reference Material of China, an internal standard solution of eight elements for ICP-MS analysis, included Li, Sc, Ge, Y, Rh, In, Re and Bi (CFGG-163175-02-01), was obtained from the National Standard Samples Website, and high purity nitric acid and hydrofluoric acid were purchased from Beijing Chemical Reagent Factory (Beijing, China). And their analytical methods were presented below.

### Soil digestion

The soils were freeze-dried and sieved through a 60 meshes sieve, and then 0.1 g of soil was weighed into a digestion cell. The digestion was carried out on a microwave digestion system (WX-6000, PreeKem Scientific Instruments Co., Ltd.) with a digestion solution of 7 ml of nitric acid and 3 ml of hydrofluoric acid. The program for microwave digestion was heated to 100 °C within 5 min, held at 100 °C for 2 min; then to 150 °C within 5min, and held at 150 °C for 3 min. Final to 180 °C within 5 min, and held at 180 °C for 25 min. After cooling, the digestion cell was taken out and then evaporated to near dryness at 95 °C using an electric hot plate. At last, 1 mL of nitric acid was injected into the tubes and the solution was diluted by triple-distilled water to 20 mL, then homogenized and stored at 4 °C for analysis.

### Instrumental analysis

All samples were spiked with internal standard solution, the isotope of each element and its corresponding internal standard were selected and presented in Table S5 [7]. The concentrations of metal analysis were conducted on an Agilent 7700 inductively coupled plasma-mass spectrometer (ICP-MS). The RF power, S/C temperature, and analyzer pressure were set at 1550 W, 2 °C, and 1×10^-4^ Pa, respectively. Argon was used as carrier gas and circuit flow was at 1 L/min.

**Table S5** Isotopes of heavy metals and its corresponding internal standard for ICP-MS analysis

| Element | Isotope | Internal standard | Detection limit |
| --- | --- | --- | --- |
|  |  |  | mg/kg |
| As | 75 | ^74^Ge | 0.4 |
| Cd | 111 | ^115^In | 0.09 |
| Cr | 52 | ^45^Sc | 2 |
| Cu | 63 | ^74^Ge | 0.6 |
| Hg^a^ | 202 | ^115^In | 0.011 |
| Ni | 60 | ^74^Ge | 1 |
| Pb | 208 | ^209^Bi | 2 |
| Zn | 66 | ^74^Ge | 1 |

^a^: The data of Hg was obtained from a literature[8] and other elements were obtained from the National Environmental Protection Standards of China (HJ 803-2016) [7] .

### Quality assurance and quality control (QA/QC)

The detect limitation for eight metals were in the range of 0.011 – 2 mg/kg. Moreover, solvent blanks, standard reference materials were processed three duplicates for QA/QC. As a result, the concentrations of heavy metals in blanks were not detected, the recoveries of target metals were in the range of 97.6 – 106%, and their relative deviation were < 8% for all batch treatments. In addition, each batch of 10 samples was typically checked with at least one calibration standard solution and the relative differences between the calibration curve and the checks were all within 10%.

**References**

[1] Qi H., Chen X., Du Y.-e., Niu X., Guo F., Li W. 2019 Cancer risk assessment of soils contaminated by polycyclic aromatic hydrocarbons in Shanxi, China. *Ecotoxicol. Environ. Saf.* **182**. (https://doi.org/10.1016/j.ecoenv.2019.109381) .

[2] SSY 2018. Table 6-6: Coal production in major years. Shanxi Statistics Yearbook, <http://tjj.shanxi.gov.cn/tjsj/tjnj/nj2018/indexch.htm>.

[3] CSY 2019. Table 9-1: energy production in major years. China Statistics Yearbook, <http://www.stats.gov.cn/tjsj/ndsj/2019/indexch.htm>.

[4] SSY 2018. Table 6-7: Coal consumption in major years. Shanxi Statistics Yearbook, http://tjj.shanxi.gov.cn/tjsj/tjnj/nj2018/indexch.htm

[5] CSY 2019. Table 9-2: energy consumption in major years. China Statistics Yearbook, <http://www.stats.gov.cn/tjsj/ndsj/2019/indexch.htm>.

[6] CNEMC 1990. Soil elements background values in China (in Chinese) China National Environmental Monitoring Center, China Environmental Science Press, Beijing.

[7] NEPSC 2016. Soil and sediment determination of aqua regia extracts of 12 metal elements-Inductively coupled plasma mass spectrometry (in Chinese). National Environmental Protection Standards of China, Beijing, China.

[8] Li Y. F., Chen C. Y., Li B., Sun J., Wang J. X., Gao Y. X., Zhao Y. L., Chai Z. F. 2006 Elimination efficiency of different reagents for the memory effect of mercury using ICP-MS. *J. Anal. At. Spectrom.* **21**: 94-96. (10.1039/B511367A) .
